# Fabrication of CMC-*g*-PAM Superporous Polymer Monoliths via Eco-Friendly Pickering-MIPEs for Superior Adsorption of Methyl Violet and Methylene Blue

**DOI:** 10.3389/fchem.2017.00033

**Published:** 2017-06-08

**Authors:** Feng Wang, Yongfeng Zhu, Wenbo Wang, Li Zong, Taotao Lu, Aiqin Wang

**Affiliations:** ^1^Key Laboratory of Clay Mineral Applied Research of Gansu Province, Center of Eco-Material and Green Chemistry, Lanzhou Institute of Chemical Physics, Chinese Academy of SciencesLanzhou, China; ^2^Graduate University of the Chinese Academy of SciencesBeijing, China

**Keywords:** eco-friendly Pickering emulsions, palygorskite, porous materials, adsorption, dye

## Abstract

A series of superporous carboxymethylcellulose-*graft*-poly(acrylamide)/palygorskite (CMC-*g*-PAM/Pal) polymer monoliths presenting interconnected pore structure and excellent adsorption properties were prepared by one-step free-radical grafting polymerization reaction of CMC and acrylamide (AM) in the oil-in-water (O/W) Pickering-medium internal phase emulsions (Pickering-MIPEs) composed of non-toxic edible oil as a dispersion phase and natural Pal nanorods as stabilizers. The effects of Pal dosage, AM dosage, and co-surfactant Tween-20 (T-20) on the pore structures of the monoliths were studied. It was revealed that the well-defined pores were formed when the dosages of Pal and T-20 are 9–14 and 3%, respectively. The porous monolith can rapidly adsorb 1,585 mg/g of methyl violet (MV) and 1,625 mg/g of methylene blue (MB). After the monolith was regenerated by adsorption-desorption process for five times, the adsorption capacities still reached 92.1% (for MV) and 93.5% (for MB) of the initial maximum adsorption capacities. The adsorption process was fitted with Langmuir adsorption isotherm model and pseudo-second-order adsorption kinetic model very well, which indicate that mono-layer chemical adsorption mainly contribute to the high-capacity adsorption for dyes. The superporous polymer monolith prepared from eco-friendly Pickering-MIPEs shows good adsorption capacity and fast adsorption rate, which is potential adsorbent for the decontamination of dye-containing wastewater.

## Introduction

Porous functional polymer materials have been widely concerned in both academia and industry areas because of their excellent properties and potential applications in many fields such as catalysis (Pierre et al., 2006; Zhang et al., 2008; Chan-Thaw et al., 2010; Kovačič et al., 2014), sensing (Zhao et al., 2007), separation (Zhao et al., 2007; Alexandratos, 2009; Furukawa and Yaghi, 2009; Furukawa et al., 2014; Gándara et al., 2014; Wang B. et al., 2015), adsorption (Yu et al., 2015; Zhu et al., 2016), and tissue engineering (Zhang et al., 2005; Hu et al., 2014; Viswanathan et al., 2015). So far, many approachs including gas foaming (Dehghani and Annabi, 2011; Ji et al., 2012), freeze drying (Autissier et al., 2010), self-assembly of block copolymer (Bolton et al., 2011), and micellar template (Wang W. B. et al., 2013) have been employed to prepare porous polymer materials, but it is difficult to form interconnected pores by these foaming methods. In recent years, it has been proved that high internal phase emulsions (HIPEs) are effective soft templates of preparing multi-porous polymer materials (Kimmins and Cameron, 2011). The polymer materials presenting interconnected porous structure could be obtained by initiating the polymerization of monomers in the continuous phase and then removing the internal phase (Silverstein, 2014). However, the O/W HIPEs are typically concentrated emulsions containing more than 74% internal phase (Huš and Krajnc, 2014). Therefore, the formation of such emulsion requires a large amount of toxic organic solvents (i.e., toluene, *n*-hexane, *p*-xylene, and liquid paraffin) as the dispersed phase, and surfactant as the stabilizer. The use of large amount of organic solvents and surfactants not only increased the cost, but also causes serious environmental pollution. In addition, the pore wall of the materials prepared from HIPEs is thin, which affects the strength of the porous materials.

Pickering high internal phase emulsions (Pickering-HIPEs) are emulsions stabilized with nanoparticles, which have recently been employed for preparing porous polymer materials (Ikem et al., 2010a, 2011; Kovačič et al., 2012). In Pickering-HIPEs, nanoparticles are present at the oil/water (O/W) interface, evenly packed around the emulsion droplets to form a dense protective layer, so that the size of Pickering emulsion droplets are larger than the dropletes stabilized with surfactants (Xu et al., 2015). There are gaps between the accumulated emulsion droplets, which can accommodate the monomers to form the monomer layer. After the monomers were polymerized, a thicker polymer layer was formed on the outer layer of the emulsion droplets. The emulsion droplet as a support prevented the coalescence of monomers and collapse of polymer layer (Kimmins and Cameron, 2011). After removal of the emulsion droplets, a porous polymer material having solid pore wall was formed. Since the Pickering emulsion is stabilized with nanoparticles, the amount of surfactants can be greatly reduced as compared to the conventional HIPEs stabilized with surfactants alone. In addition, the nanoparticles used to stabilize the emulsion can participate in the polymerization reaction to improve the structure and strength of polymer network (Haibach et al., 2006; Brun et al., 2011).

Although the use of HIPEs template method for preparation of porous polymer has made great progress (Kimmins et al., 2012; Kovačič et al., 2012; Mert et al., 2013), there are still two problems that need to be addressed. First, continuous phase currently used to prepare Pickering emulsion is a toxic organic solvent, and the nanoparticles used to stabilize Pickering emulsion are mainly synthetic nanoparticles. Second, the polymerization of monomers in the Pickering-HIPEs stabilized with only nanoparticles usually produces porous polymer materials with a super closed-cell pore, typically between 200 and 700 μm, which limited the permeability of the porous material (Xu et al., 2015). So, it would be a feasible approach to reduce the volume of dispersion phase and replace the potentially toxic organic solvents and nanoparticles with naturally available ones are the effective approaches to solve the problems. Therefore, reducing the volume of the dispersed phase, replacing toxic organic solvents with non-toxic oil phase, replacing synthetic nanoparticless with natural ones, and introducing nanoparticles and surfactants simultaneously to reduce the pore sizes (Ikem et al., 2010b, 2011) would be feasible ways to overcome these problems.

Unlike Pickering-HIPEs, formation of Pickering-medium internal phase emulsions (Pickering-MIPEs) requires less organic solvents, and the size of the droplets in Pickering-MIPEs can be adjusted by changing the content of nanoparticles or introducing appropriate amount of surfactant. So, Pickering-MIPEs are more advantageous and potential than Pickering-HIPEs in the preparation of porous materials. Menner et al. (2007) reported the porous polyFoams prepared from the Pickering-MIPEs composed of the carbon nanotube as stabilizer and the mixture solvents of styrene and poly(ethyleneglycol) dimethacrylate (PEGDMA) (*V/V* = 50:50) as dispersed phase. It has been proved that the Pickering-MIPEs are highly effective template to produce an interconnected porous structure. However, there has been no report on the preparation of porous polymer monolith using eco-friendly Pickering-MIPEs prepared using edible oil as dispersed phase and nanoscale clay mineral as stabilizer.

Palygorskite (Pal) is a natural one-dimensional nanorod-shaped hydrated Mg-rich silicate clay mineral, with the diameter of about 20–40 nm and length of 0.5–5 μm (Galán, 1996; Wang et al., 2014). It has been widely used in adsorption (Sarkar et al., 2012; Wang et al., 2015a), colloid (Xu et al., 2013), carriers (Papoulis et al., 2010), reinforcing (Huang et al., 2012; Ruiz-Hitzky et al., 2013) and other fields. It has been reported that the water-in-oil (W/O) emulsion is usually susceptible to be stabilized by hydrophobic particles, while oil-in-water (O/W) emulsion is normally stabilized by hydrophilic particles (Zheng et al., 2013). Pal is rich in Si-OH groups on surface, so it is hydrophilic and can stabilize O/W emulsion, without any modification (Pan et al., 2013). The combination of Pal and non-toxic and low-cost edible oil (for replacing toxic organic solvents) is expected to form an eco-friendly Pickering-MIPEs for the synthesis of polymer monoliths having interconnected pore structure.

In order to develop eco-friendly adsorbents with rich functional groups, better porous structure and excellent adsorption capability, we used edible oil as the dispersed phase and natural Pal nanorods as particle stabilizers to prepare Pickering-MIPEs, and then a series of polymer monoliths were prepared by free-radical grafting polymerization of CMC and AM in the emulsions. The effects of Pal dosage, AM dosage, and co-surfactant T-20 on the pore structure and adsorption properties of the monoliths were investigated, and the adsorption behaviors for MV and MB dyes were evaluated systematically.

## Experimental

### Materials

Pal was from Huangnishan Mine in Xuyi county of Jiangsu province, China, which was crushed and passed through a 200-mesh sieve. Sodium carboxymethylcellulose with the characteristic viscosity of 300–800 mpas (1% aqueous solution) (C.P. grade), and acrylamide (AM, C.P. grade) was purchased from Shanpu Chemical Factory (Shanghai, China). Ammonium persulfate (APS, A.R. grade), *N,N,N*′*N*′-tetramethylethylenediamine (TEMED, A.R. grade), methyl violet (MV), and methylene blue (MB) were purchased from Sinopharm Chemical Reagent Co., Ltd. (Shanghai, China). *N, N*′-methylene-*bis*-acrylamide (MBA, C.P. grade) was purchased from Yuanfan additives plant (Shanghai, China). Tween-20 (T-20, A.R. grade) was purchased from BASF Corporation (Germany), and edible plant oil was purchased from Yihaijiali Arawana Edible Oil Company, China. 3A molecular sieve was purchased from Molsion Molecular Sieve Co., Ltd. (Shanghai, China). Other reagents were of analytical grade and all solutions were prepared using deionized water.

### Preparation of pickering-MIPEs

Certain amounts of Pal and T-20 (contents of T-20 or Pal were corresponding to the volume of dispersed phase) were dispersed in 10 mL deionized water under mechanical agitation, and then 10 mL of vegetable oil was added, and the mixture was stirred at 10,000 rpm for 5 min using a GJD-B12K high-speed emulsifier to form Pickering-MIPEs. The type of the emulsions was determined by the pendant-drop method using deionized water and toluene as medium. It was observed that the emulsion droplets could be dispersed in deionized water but stay round in toluene, indicating the as-prepared Pickering-MIPEs are O/W emulsions.

### Preparation of porous CMC-*g*-PAM/Pal monoliths

**Figure 1:**
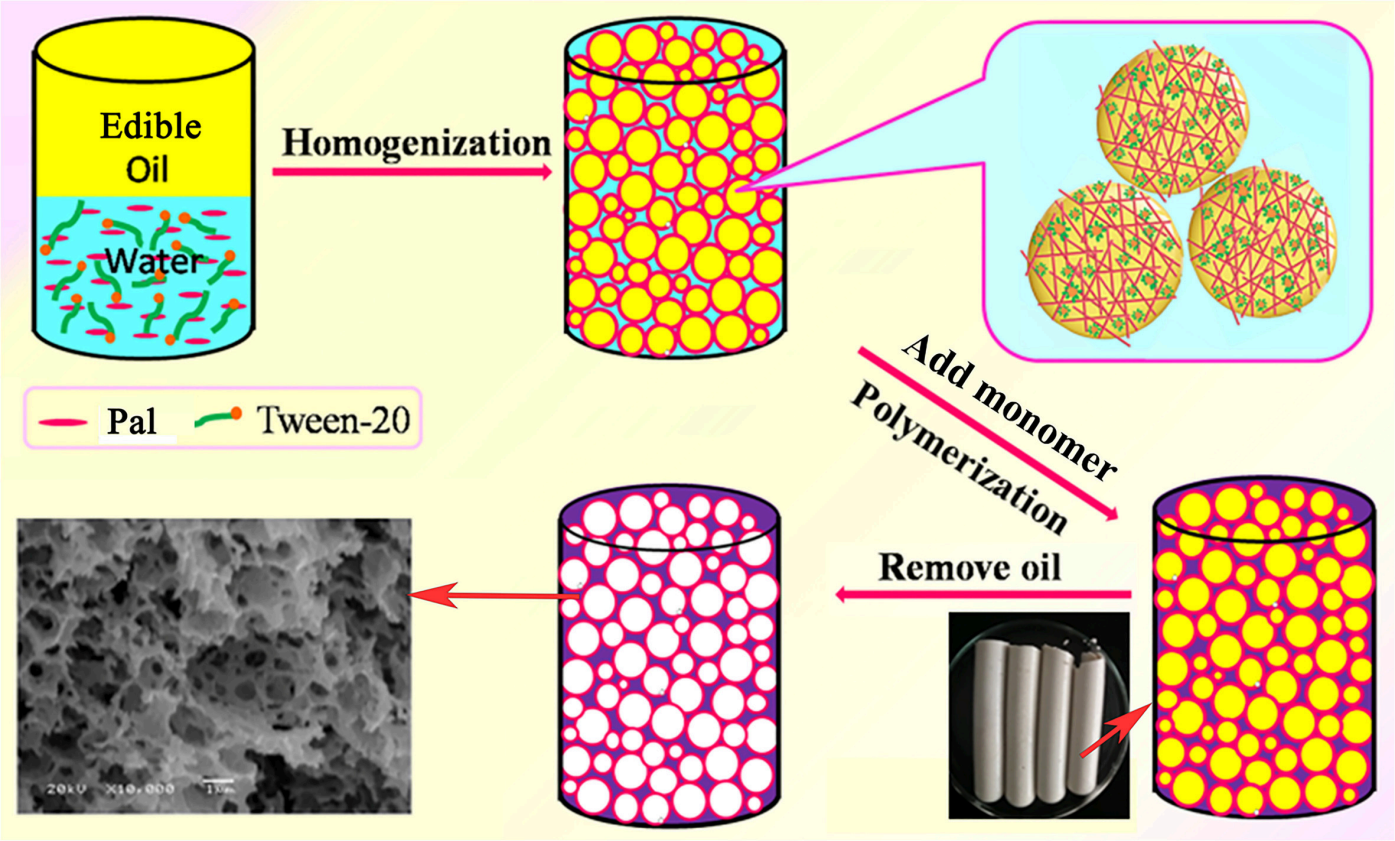
The preparation progress of the porous monoliths.

First, the Pickering-MIPEs containing 0.1 g of CMC, 0.31 g of MBA, 3% of T-20 and different amounts of Pal were firstly prepared by dissolving CMC and MBA into the continuous phase of emulsion. Then, different amounts of AM, 91 mg of APS and 0.1 mL of TEMED were dissolved in the emulsion by vigorous mechanical agitation. The Pickering-MIPEs containing monomer, initiator and crosslinker were transferred to test tubes, and the test tubes were sealed and soaked in a 65°C water bath for 24 h to finish the polymerization reaction. The cylindrical gel product was cut into small pieces, and washed sequentially with *n*-hexane and ethanol *via* a Soxhlet extractor. The washed product was immersed in 0.5 mol/L of the NaOH solution in 70% ethanol (*V*/*V*) for 24 h to transform amide groups into carboxyl groups. The hydrolyzed product was washed with water/ethanol (*V*_water_/*V*_alcohol_ = 3/7) mixture solvents to remove the undesirable residues. Finally, the product was sufficiently dehydrated with absolute ethanol in the presence of 3A molecular sieve, and then dried under vacuum at 60°C for 8 h. The feed composition of reactants, the codes of the porous monoliths and the corresponding pore structure parameters are listed in Table 1, and the preparation process of the polymer monoliths is shown in Figure 1.

**Table 1:** Feed composition for preparing the monoliths, and the average pore size (D) and surface area (A) of the as-prepared monoliths.

**Codes**	**T-20 (%)**	**Pal (%)**	**AM (mmol)**	**D_m(macro)/μm_**	**D_m(pore throat)/μ*m*_**	**A_(macro)/μm_^2^**	**A_(pore throat)/μm_^2^**
PA-11	0	11	20	46.51	–	–	–
PAT-7	3	7	20	1.44	0.41	360.91	233.90
PAT-9^a^	3	9	20	1.47	0.39	399.35	297.88
PAT-11	3	11	20	1.48	0.43	405.78	311.81
PAT-13	3	13	20	1.41	0.40	401.47	323.77
PAT-15	3	15	20	1.31	0.42	408.31	321.48
PM-10	3	9	10	–	–	–	–
PM-20^a^	3	9	20	1.47	0.39	399.35	297.88
PM-30	3	9	30	1.30	0.53	343.83	231.68
PM-40	3	9	40	1.37	0.43	307.83	194.47
PM-50	3	9	50	1.41	0.45	308.15	146.00

a *PAT-9 is the same with PM-20*.

### Characterizations

The surface morphologies of the polymer monoliths were observed using a JSM-6701F field emission scanning electron microscope (SEM, JEOL, Japan) after the sample was coated with gold film. FTIR spectra were measured using a Nicolet NEXUS FTIR spectrometer (U.S.A.) in the wavenumber range of 4,000–400 cm^−1^ after the samples were pressed as KBr pellets. The size distribution of pores and pore throat were calculated using Image-Pro Plus 6.0 software by statistically counting the size of 100 pores.

### Evaluation of adsorption properties

The adsorption properties of the polymer monoliths were evaluated through batch adsorption experiment. Typically, 20 mg of the monolith was mixed with 50 mL of the aqueous solutions of MV or MB (100–1,000 mg/L), and then the mixture was shaken on a thermostatic shaker (THZ-98A) at 120 rpm and 30°C for 120 min to achieve adsorption equilibrium. After that, the monolith was separated from the solution by filtering through a 300-mesh sieve. The absorbance of MB and MV solutions was measured at the maximum absorption wavelength of 664 and 583 nm, respectively using a UV–visible spectrophotometer (UV-3010, HITACHI, Japan), and the concentration of MB and MV was calculated according to the standard curve method. The adsorption capacities of the polymer monoliths could be calculated according to Equation (1):

(1) $$\begin{array}{r} q_{e}=\left(C_{0}-C_{e}\right)\times V/m \end{array}$$

where, *q*_*e*_ (mg/g) is the ability of the unit mass of adsorbent to adsorb dyes; *C*_0_ and *C*_*e*_ (mg/L) are the concentrations of MV and MB solutions before and after adsorption, respectively. *V* (L) is the volume of dye solutions used for adsorption test. *m* (mg) is the mass of the adsorbent used.

The effect of pH on the adsorption was studied by evaluating the adsorption capacities of monolith at the initial MB or MV concentration of 600 mg/L and different pH conditions (from 2 to 10). The pH of solution was adjusted with 0.1 mol/L HCl or NaOH solutions. The effect of initial MB or MV concentrations on adsorption was studied by determining the adsorption capacities at different initial concentrations from 100 to 1,000 mg/L (pH 6). The effect of contact time on adsorption was studied by evaluating the adsorption capacities at different time intervals from 5 to 120 min (initial concentration, 200 mg/L; pH 6). The removal efficiency was evaluated using 5 mg of the monolith adsorbent and different volumes of dye solution (initial concentration, 200 mg/L). All adsorption experiments were repeated three times, and the averages were reported in this paper.

The reusability of the monolith was evaluated by determining the adsorption capacities of the adsorbent regenerated by desorption process. Typically, 20 mg of the monoliths were fully contacted with 50 mL of MB or MV solutions with the initial concentration of 600 mg/L to reach adsorption equilibrium, and then the dye-loaded monoliths was immersed into 30 mL of 0.5 mol/L HCl solution for 2 h, followed into 0.5 mol/L NaOH solution for 24 h. Finally, the polymer monoliths were sufficiently washed with deionized water to neutral, and then used for the next adsorption cycle. The above procedure was repeated for five times to evaluate the reusability of the polymer monolith adsorbent.

## Results and discussion

### Pickering MIPEs stabilized with Pal nanorods

**Figure 2:**
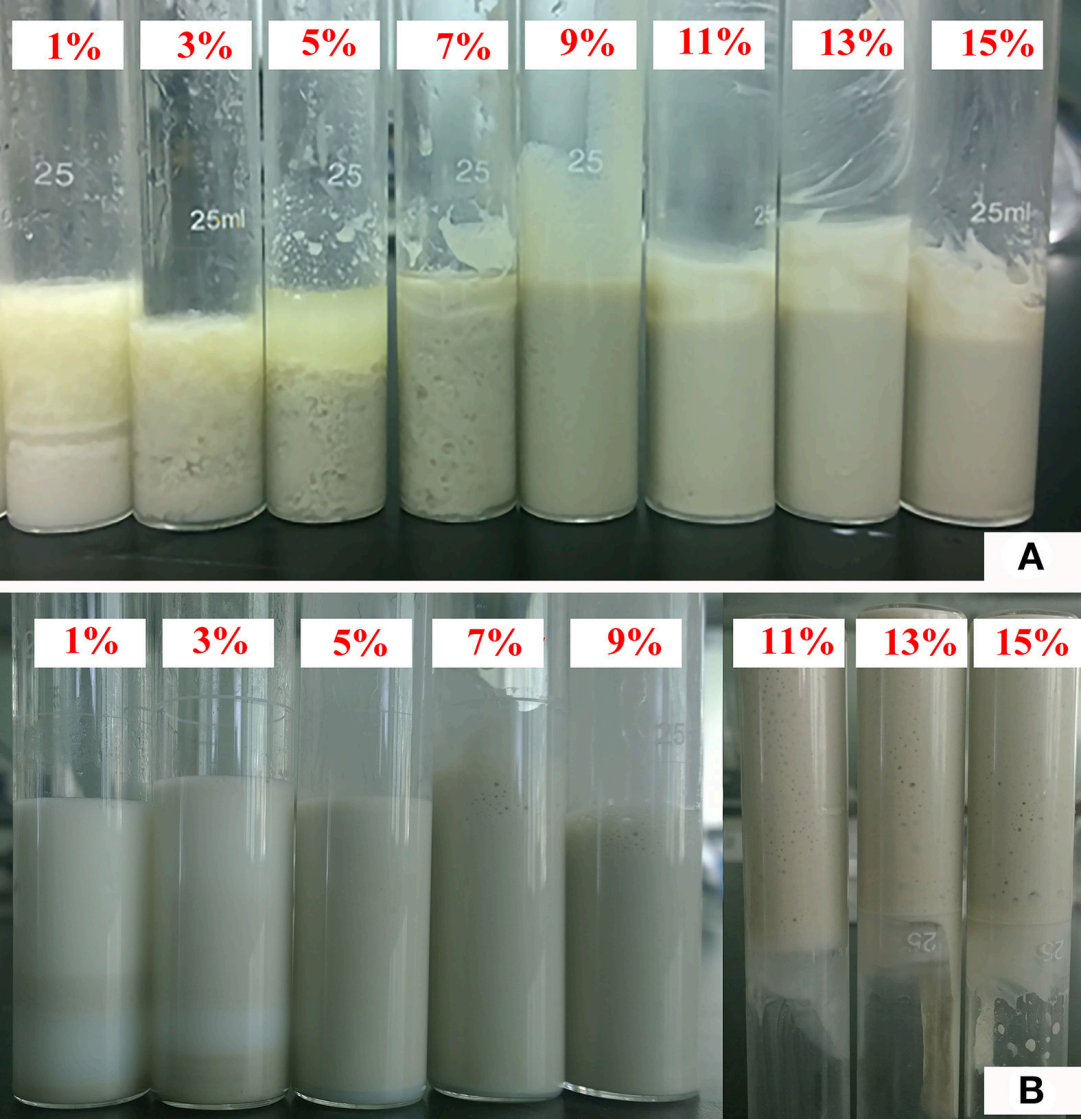
Digital photos of Pickering-MIPEs (*V*_water_/*V*_oil_ = 1:1) stabilized with **(A)** different amounts of Pal (no surfactant), and **(B)** 3% T-20 and different amounts of Pal.

Pal content has a significant influence on the composition and characteristics of Pickering emulsions. As shown in Figure 2A, when Pal content is less than 9%, the emulsion creaming phenomenon occurred; and when the Pal content is 9–14%, a stable emulsion was formed. Similar to other solid particles (Binks et al., 2010; Kim et al., 2010; Zheng et al., 2013), the increase in Pal content increased the volume and stability of the emulsion. The main reason is that the increase in the number of particles can increase the interface area and form a more dense nanoparticle layer in the O/W interface, so that the emulsion becomes more stable (Zheng et al., 2014). The good stability of the emulsion also demonstrates that Pal is an ideal particle stabilizer to prepare O/W Pickering-MIPEs.

**Figure 3:**
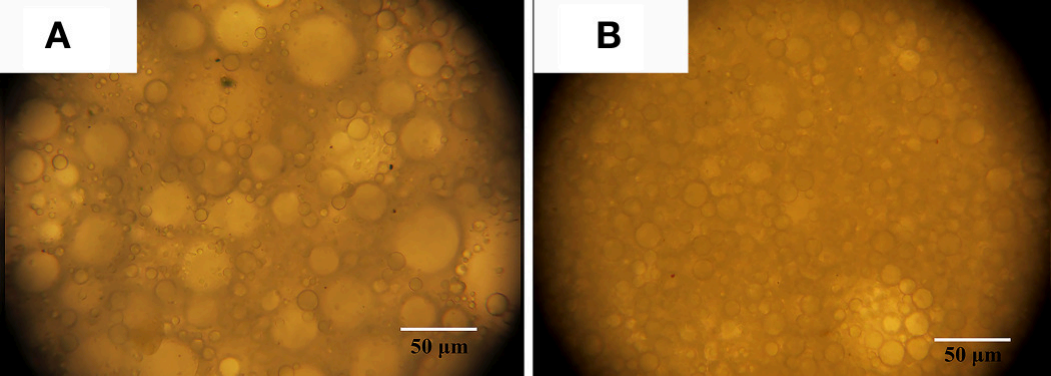
Optical photos of Pickering MIPEs stabilized by **(A)** 11% Pal without surfactant; **(B)** 11% Pal and 3% Tween-20 surfactant.

It was observed from Figure 2B that the emulsion becomes more stable with the increase in Pal content. When the Pal content is 9–14%, the stable Pickering-MIPEs were obtained. As reported previously (Zou et al., 2013), the emulsion droplets stabilized with only particles are bigger than that stabilized with particles and surfactants simultaneously, which are consistent with our experimental results, as shown in Figure 3. Pal has rod-shaped crystal morphology, which make it difficult to completely cover the outer surface of the droplets. So that the T-20 surfactant can be adsorbed on the O/W interface that are not covered by Pal, which reduced the interfacial tension and broken larger droplets as smaller ones under the action of mechanical agitation (Ikem et al., 2010b).

### Structure characteristics of porous monoliths

The porous polymer monoliths were prepared by the following procedure: (i) the grafting polymerization of CMC, AM monomers and Pal in the emulsion; and (ii) removal of the internal oil phase (Figure 1). In this process, the coalescence of partially neighboring droplets, the volume shrinkage caused by polymerization of monomers, and the separation of surfactant-rich phase and polymer-rich phase contributed to the formation of open pores (Cameron et al., 1996; Xu et al., 2015).

**Figure 4:**
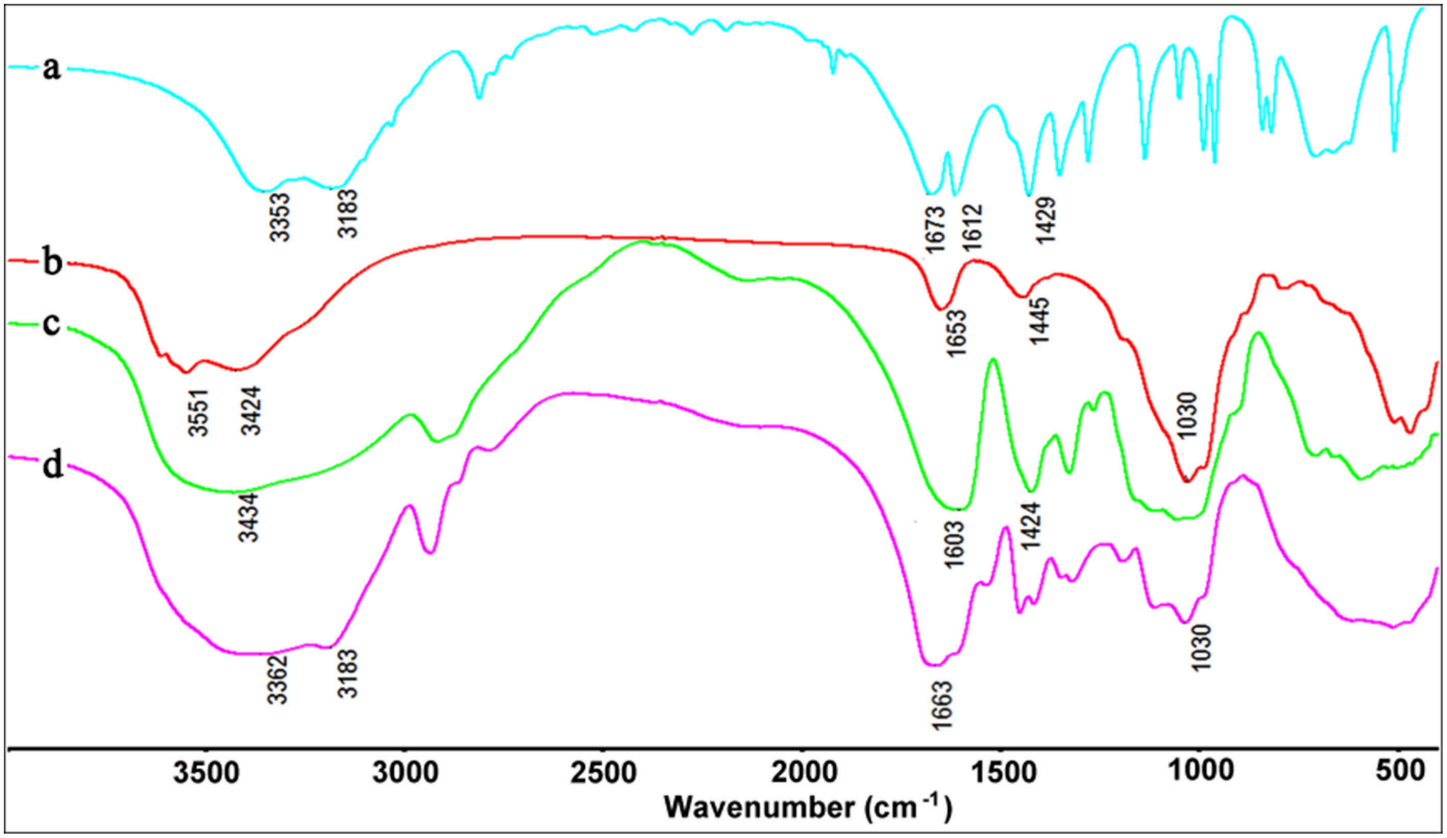
FTIR spectra of (a) AM, (b) Pal, (c) CMC, and (d) CMC-*g*-PAM/Pal monolith.

The formation of CMC-*g*-PAM/Pal was confirmed by FTIR spectra analysis. As shown in Figure 4, the absorption band of AM at 1,612 cm^−1^ (C=C stretching vibration) disappeared after forming CMC-*g*-PAM/Pal monolith. The absorption band of Pal at 1,030 cm^−1^ (the Si-O-Si asymmetric stretching vibration) appears in the spectrum of CMC-*g*-PAM/Pal, indicating there is Pal in the porous monolith. The band of Pal at 3,551 cm^−1^ [the O-H stretching vibrations of (Fe, Mg)O-H and (Al, Mg)O-H)] (Wang et al., 2015b), and the absorption band of CMC at 3,434 cm^−1^ (the O-H stretching vibration of CMC) were overlapped, which appear at 3,362 cm^−1^ in the spectrum of CMC-*g*-PAM/Pal. The absorption band of Pal at 1,653 cm^−1^ (H-O-H bending vibration of zeolite and bonding water), the band of AM at 1,673 cm^−1^ (C = O stretching vibration) and the band of CMC at 1,603 cm^−1^ (asymmetric stretching vibration of carboxylate) were overlapped and appeared at 1,663 cm^−1^, indicating that the AM monomer was grafted onto CMC (Xiao et al., 2015).

**Figure 5:**
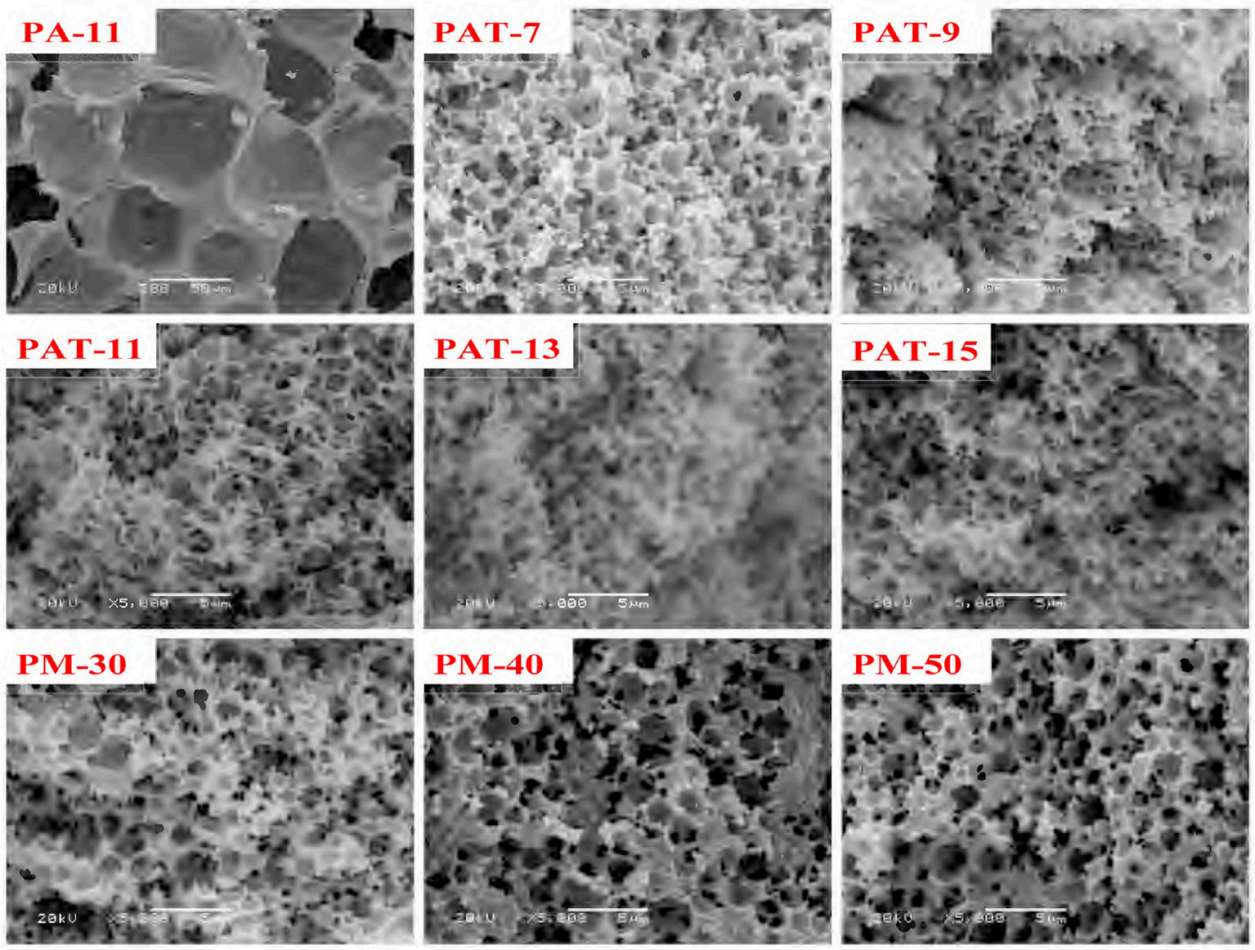
SEM images of the porous monoliths.

The pore characteristics of the monoliths prepared from Pal-stabilized Pickering emulsion (without T-20) were studied by SEM analysis, as shown in Figure 5. The as-prepared monolith shows closed-cell macropore with the average pore size (D_m_ (macro)) of 46 μm (Table 1). It has been reported that when small amount of surfactant was used as a co-stabilizer, the closed-cell pores formed from Pickering-MIPEs could be transformed as interconnected pores (Horozov, 2008). Our research results also show that highly interconnected small pores were formed in the monolith by introducing 3% of T-20 as the co-stabilizer (Figure 4). It was shown from Table 1 that the average sizes of macropores and pore throat of the monolith are 1.48 and 0.43 μm, respectively.

**Figure 6:**
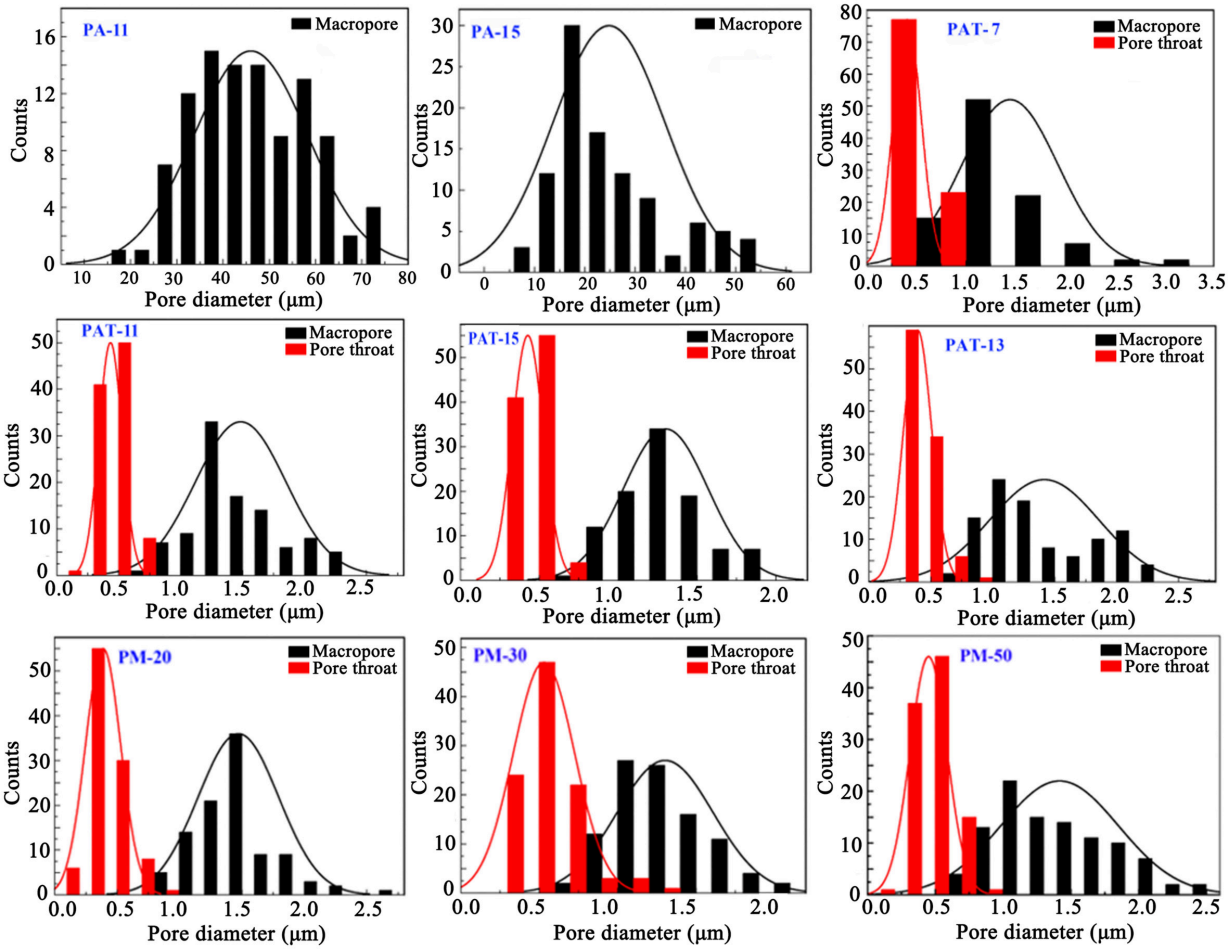
The pore diameter distribution of macropores and pore throat of the porous monoliths prepared at different conditions.

In general, macropore is an ideal reservoir of ions because it could reduce the diffusion distances; while pore throat is related to the surface area of porous materials (Wei et al., 2015). As shown in Figure 6, the size distribution of macropores in the polymer monolith is from 0.5 to 2.5 μm, and the size distribution of pore throat is from 0.2 to 1 μm. The well-defined pores facilitate to the contact of dye molecules with the monoliths, and thus reduce the mass-transfer resistance and enhance the adsorption rate.

After introducing 3% of T-20, the pore wall becomes thinner and the porosity increased (Figure 5, Table 1). When Pal content increased to 9%, the surface areas of macropore and pore throat increased. When Pal content increased to 15%, the surface area of the monolith is in a narrow range, and interconnected small pores were formed (Figure 5, PAT-9, PAT-11, PAT-13, and PAT-15). At the high dosage of Pal, more particles accumulate on the O/W interfaces to form dense nanoparticle layer. Thus, the thin films among the droplets make sure the formation of interconnected pore structure in the polymer monolith (Ikem et al., 2008; Zheng et al., 2014). The effect of Pal content on the pore size distribution of the monoliths was shown in Figure 6. It was revealed that the size distribution of macropores and pore throat are narrow in all the porous monoliths.

The AM dosage has a significant effect on the structure and morphologies of the monoliths. As shown in Figure 5, no pores were observed in the monolith when the AM dosage is 10 mmol. However, when the AM dosage increases to 20 mmol, the hierarchically structured pores were formed in the monolith. As the AM dosage is further increased, the pore throats and macropores were reduced and the pore walls become thicker. The main reason is that the increase in AM dosage makes the monomer layer between neighboring droplets become thicker, so that the pore wall becomes thicker and can be remained well. In this case, the polymer monolith with interconnected pores was formed by the polymerization reaction of AM monomers in the Pickering-MIPEs.

It has been reported that nanoparticles can serve as bridging or cross-linking points in polymer network to improve its mechanical property (Gao et al., 2014). So, there are similar interactions between the Si-OH groups of Pal and the -OH, -NH_2_, -COO^−^, amide groups on polymer chains in the porous monoliths (Darder et al., 2005). This means that Pal is not only a stabilizer of emulsion, but also an inorganic composite component of the polymer material. As a whole, the superporous polymer monoliths can be prepared by the simple free-radical polymerization in the Pickering-MIPEs stabilized by only Pal particle or the mixture of Pal and surfactant T-20.

### Evaluation of adsorption properties

#### Effect of pH values on adsorption

**Figure 7:**
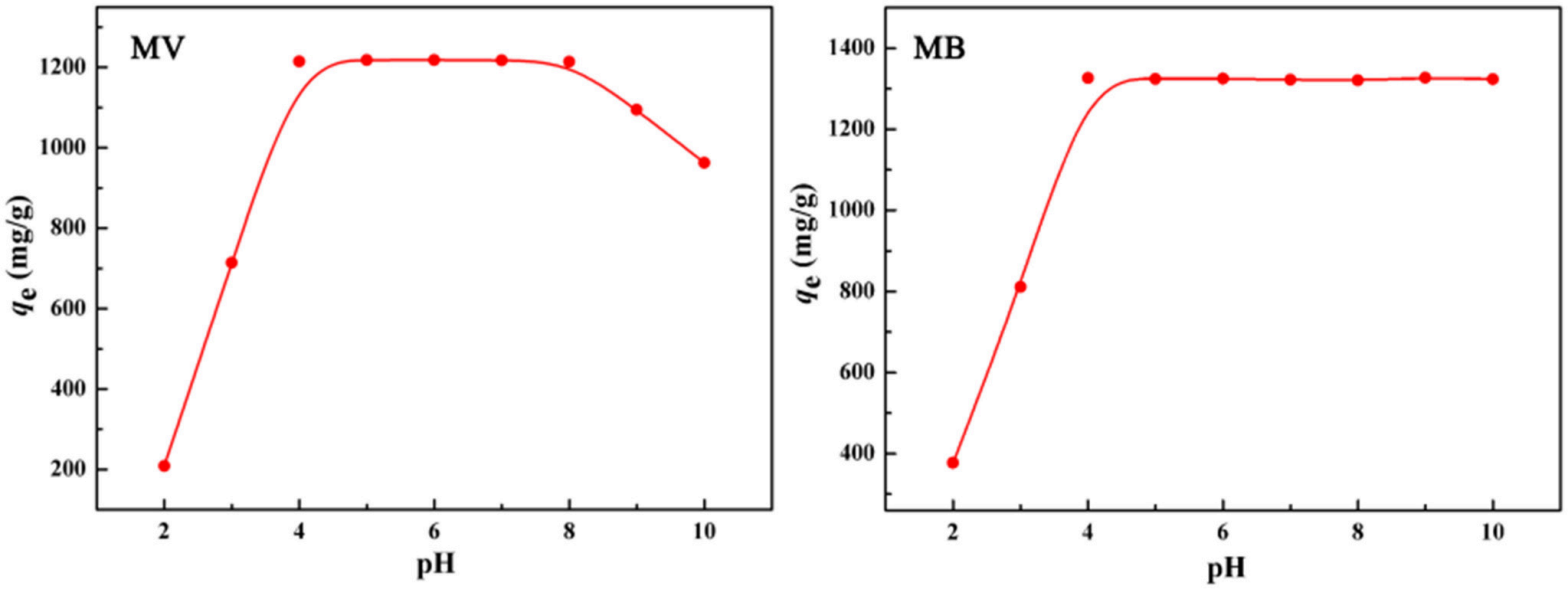
Effect of pH on the adsorption capacity of the porous monolith for MV and MB, respectively. Adsorption conditions: *C*_0_, 600 mg/L; dosage of adsorbent, 0.40 g/L.

As shown in Figure 7, the adsorption capacity of the polymer monolith for MV increased with the increase in pH, almost remains constant at pH 4.0–8.0, and then rapidly deceased when the pH was higher than 8. However, adsorption capacity for MB almost keeps constant at a wide pH range from 4 to 10. The rapid increase of adsorption capacity with increasing the pH from 2 to 4 is due to the change of chemical groups. Under acidic condition, the -COO^−^ groups in the polymer monolith were converted to the -COOH groups that have relatively weaker electrostatic or bonding interaction with dye molecules than -COO^−^ groups, and so the adsorption capacity is low at acidic condition. With the increase in pH, the -COOH groups gradually convert to -COO^−^ groups that has strong affinity with positively charged dye molecules, which make the adsorbent able to adsorb more dye molecules. However, the amines groups of MV de-protonate at pH > 8, so that the adsorption capacity of the monolith for MV begin to decrease at pH > 8 (Tian et al., 2016a). From above discussion, it can be concluded that the electrostatic and complexation association between negatively charged -COO^−^ groups and positively charged MB^+^ or MV^+^ mainly contribute to the adsorption.

#### Adsorption isotherms

**Figure 8:**
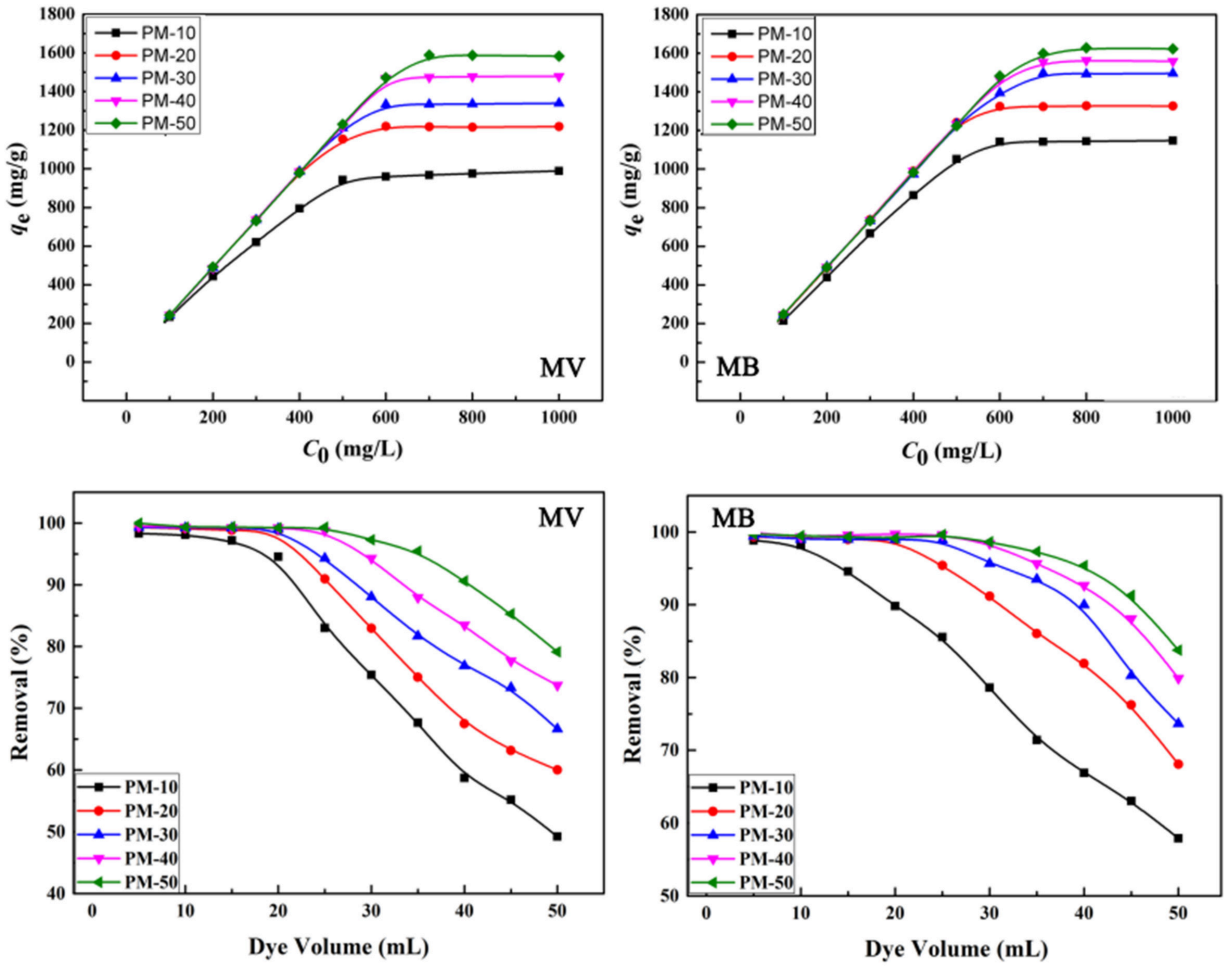
Effect of initial concentrations on the adsorption capacity and removal efficiency of the monoliths for MV and MB. Adsorption experimental conditions: dosage of adsorbent, 0.4 g/L; pH, 6; removal experimental conditions: *C*_0_, 200 mg/L; pH, 6.

The maximum adsorption capacity and removal efficiency of the polymer monoliths for MB and MV were evaluated here. As is shown in Figure 8, the adsorption capacity increased with the increase in the initial concentration of dyes, until adsorption equilibrium was achieved. The PM-50 monolith exhibits the maximum adsorption capacities of 1,585 and 1,625 mg/g for MV and MB, respectively. The amount of AM has a positive effect on improving the adsorption capacity, because the number of functional groups for holding dyes increased with increasing the amount of AM.

In order to study the adsorption process (Zhang et al., 2014), the adsorption isotherms were fitted with two classical models: Langmuir (Equation 2) and Freundlich isotherm models (Equation 3).

(2) $$\begin{array}{r} \frac{C_{\text{e}}}{q_{\text{e}}}=\frac{1}{q_{\text{m}}b}+\frac{C_{\text{e}}}{q_{\text{m}}} \end{array}$$

(3) $$\begin{array}{r} \log q_{\text{e}}=\log K+\left(1/n\right)\log C_{\text{e}} \end{array}$$

Here, *C*_*e*_ (mg/L) is the concentration of dye solution after adsorption equilibrium; *q*_*m*_ and *q*_*e*_ (mg/g) are the amount of dyes adsorbed by unit mass of adsorbent at time *t* and equilibrium, respectively. *b* (L/mg) is the Langmuir constant related to the affinity of binding sites, *K* is the Freundlich constant related to adsorption capacity, and *n* is the Freundlich constant related to index of adsorption intensity or surface heterogeneity. The adsorption isothermal parameters and linear correlation coefficients (*R*^2^) can be calculated by the fitting results shown in Tables S1, S2. It was found that the theoretical adsorption capacities calculated by the Langmuir model are close to the experimental values, and the *R*^2^ values calculated using the Langmuir isotherm model (*R*^2^ > 0.99) are higher than that calculated by Freundlich model. This indicates the adsorption process obeys Langmuir model, and the dyes were adsorbed onto the monolith by single layer adsorption (Wang J. et al., 2013).

The porous polymer monoliths also have higher adsorption removal efficiency toward dye molecules. As shown in Figure 8, the removal ratio of the monoliths for MB and MV increased as increasing the amount of AM monomers. All monoliths (PM20, PM30, PM40, and PM50) have a high ability to remove dye molecules with the removal ratio higher than >99%, when the volume of dye solution is less than 25 mL. It can be seen from Table 2 that the *q*_m_ values of PM-50 reached 1,588 and 1,628 mg/g for MV and MB, respectively, which are much higher than that of the adsorbents reported previously.

**Table 2:** Comparison of adsorption capacities (*q*_m_, mg/g) of various adsorbents for MB and MV.

**Adsorbents**	**MB**	**MV**	**References**
3D Tannic acid-graphene hydrogel	195	–	Luo et al., 2016
Porous Carbon Monoliths	127	–	He et al., 2013
Bimodal meso/macro-porous silica microspheres	84	–	Ataei-Germi and Nematollahzadeh, 2016
Magnesium Silicate/Reduced Graphene Oxide Nanocomposite	433	–	Gui et al., 2014
Sewage Sludge Based Granular Activated Carbon	132	–	Liu et al., 2013
Clinoptilolite	–	71	Hernández-Montoya et al., 2013
Erionite	–	84	Hernández-Montoya et al., 2013
Ethylenediaminetetraacetic acid-cross-linked-β-Cyclodextrin	84	114	Zhao et al., 2015
Magnetic GO/poly(vinyl alcohol) composite gels	271	221	Cheng et al., 2015
Hydrolyzed polyacrylamide grafted Xanthan Gum and incorporated nanosilica	498	379	El-Sayed, 2011
Superporous monolith	1,625	1,585	This work

#### Adsorption kinetics

**Figure 9:**
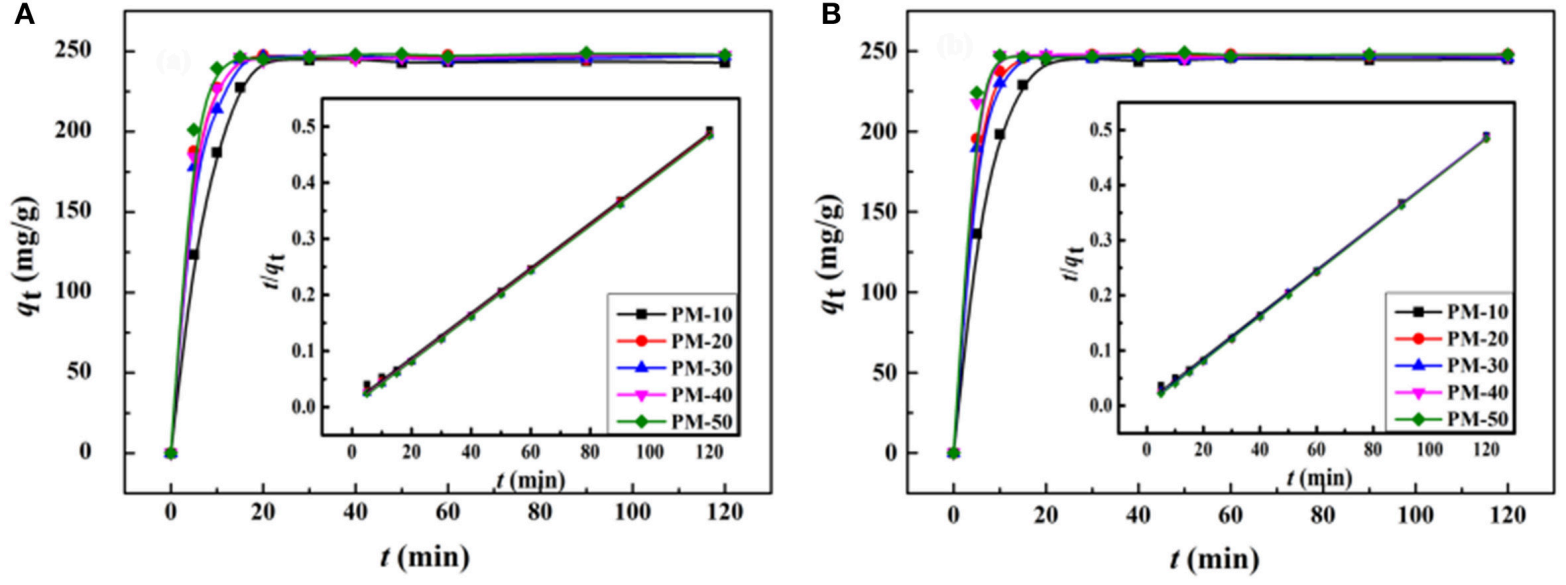
Kinetic curves of the porous monolith for adsorption of **(A)** MV and **(B)** MB. Adsorption experimental conditions: *C*_0_, 200 mg/L; dosage of adsorbent, 0.80 g/L; pH, 6.

Figure 9 shows the adsorption kinetic curves of the monoliths for MV and MB. As can be seen, the adsorption rate of the porous monoliths for dyes is fast, and the adsorption equilibrium can be reached within only 15 min. Among the five monoliths, PM-50 monolith shows the optimal adsorption capacity and rate. The main reason is that there are interconnected pores in PM-50, which make more carboxyl groups (the binding sites for dye molecules) exposed, and thus the accessibility of the adsorbent toward dyes was improved (Zheng et al., 2013; Zhu et al., 2016).

In order to examine the dynamic adsorption process, the adsorption kinetic curves were fitted by pseudo-first-order (Equation 4) (Langergren, 1898) and pseudo-second-order (Equation 5) (Ho and McKay, 1999) kinetic models.

(4) $$\begin{array}{r} \log\left(q_{\text{e}}-q_{\text{t}}\right)=\log q_{\text{e}}-\left(\frac{k_{1}}{2.303}\right)t \end{array}$$

(5) $$\begin{array}{r} \frac{t}{q_{\text{t}}}=\frac{1}{\left(k_{2}\times q_{\text{e}}^{2}\right)}+\frac{t}{q_{\text{e}}} \end{array}$$

Here, *q*_*t*_ and *q*_*e*_ are the amounts of dyes adsorbed by unit mass of adsorbents at time *t* and equilibrium, respectively. *k*_1_ (min^−1^) and *k*_2_ (g/(mg min)) are the adsorption rate constants of the pseudo-first-order and the pseudo-second-order models, respectively, which can be calculated from the slope and intercept of the plots of log (*q*_e_ − *q*_t_) vs. *t* and *t*/*q*_t_ vs. *t*, respectively. The are listed in. From the calculated adsorption kinetic parameters shown in Tables S3, S4, it can be seen that the linear correlation coefficients (*R*^2^) calculated by pseudo-second-order kinetic model are higher than 0.999; while the adsorption capacities calculated by pseudo-second-order kinetic model (*q*_e_, _cal_) are much close to the experiment values (*q*_e_, _exp_). This indicates that the adsorption process follows the pseudo-second-order kinetic model, which was controlled mainly by a chemical adsorption process (Zhou et al., 2014).

#### Adsorption mechanism

**Figure 10:**
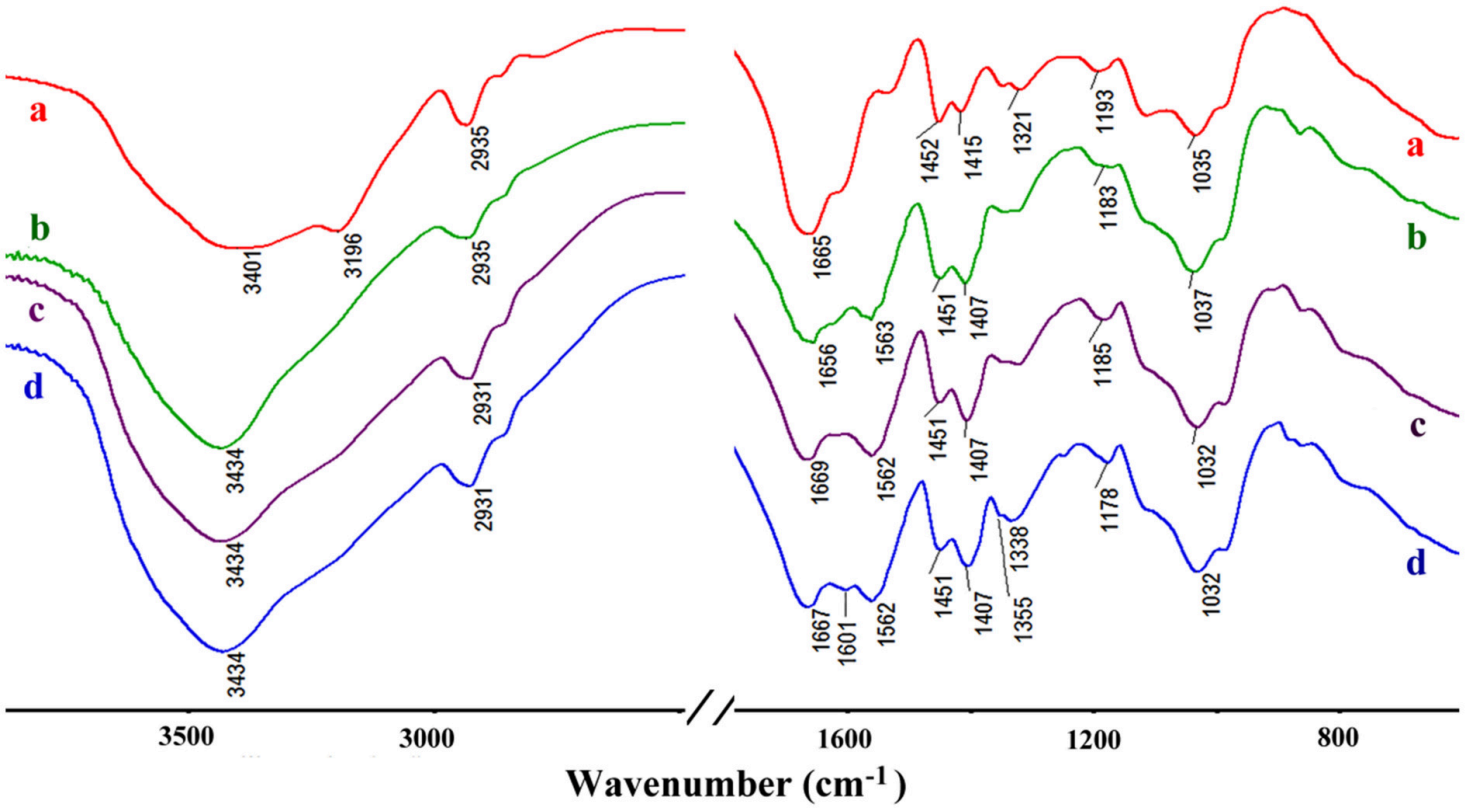
The FTIR spectra of CMC-*g*-PAM/Pal (a), hydrolytic CMC-*g*-PAM/Pal (PM-50) (b), and the PM-50 after adsorption of MV (c), and MB (d).

Figure 10 shows the FTIR spectra of CMC-*g*-PAM/Pal and PM-50 before and after adsorption of MV and MB. As shown in Figure 10a, the broad band at 3,401 cm^−1^ is assigned to the characteristic absorption of O-H and free -NH_2_, and the weaker band at 3,196 cm^−1^ is ascribed to the -NH_2_ groups in CMC-*g*-PAM/Pal. After hydrolysis, the characteristic absorption band of -NH_2_ groups disappeared (Figure 10b), and the strong absorption band at 3,434 cm^−1^ (the O-H stretching vibration) appeared. The characteristic absorption band of the C = O in amide at 1,665 cm^−1^ weakened, and the asymmetric and symmetrical stretching vibration of -COO^−^ groups at 1,563 and 1,407 cm^−1^, respectively, appeared in the spectrum of PM-50, indicating most of amide was converted to carboxylate groups (Ghorai et al., 2014). After adsorption of dyes, the absorption bands of -COO^−^ groups at 1,563 and 1,407 cm^−1^ strengthened, indicating that complexation occurred between -COO^−^ and MV or MB. The O–H stretching vibration at 3,434 cm^−1^ also strengthened, due to the H-bonding and electrostatic interaction between -OH and MV or MB molecules. The new absorption bands of MB at 1,601 cm^−1^ (the C-C stretching vibration) and 1,355 cm^−1^ (the C-N symmetric stretching vibration) were observed in the spectrum of MB-loaded adsorbent (Figure 10d), which confirmed the adsorption of dyes onto the adsorbent. It can be confirmed that the electrostatic attractions and complexation interaction between the functional groups in the monolith and MV or MB molecules mainly contribute to the adsorption of dyes (Tian et al., 2016b).

#### Regeneration and reusability

**Figure 11:**
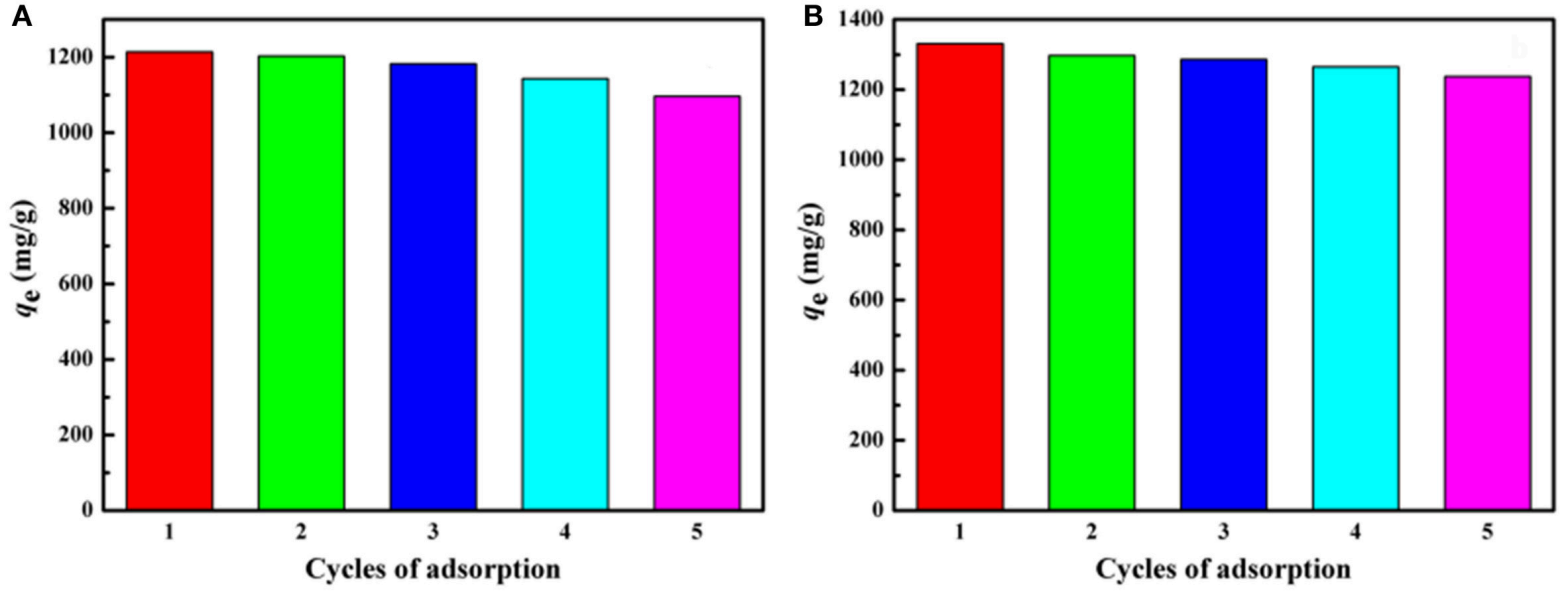
Adsorption capacities of the porous monolith for **(A)** MV and **(B)** MB after regenerated five times. Adsorption experimental conditions: *C*_0_, 600 mg/L; dosage of adsorbent: 0.40 g/L; pH, 6.

The regeneration capability of the polymer monoliths has been evaluated by adsorption-desorption process. As shown in Figure 11, the adsorption capacity for both MV and MB only decreases a little after five consecutive adsorptions-desorption cycles. The regenerated monolith still shows high adsorption capabilities of 1,108 and 1,257 mg/g for MV and MB, respectively after reused for five times, indicating the monolith is reusable and is potential to be used for purification of dye-containing wastewater.

## Conclusions

The superporous polymer monoliths have been successfully prepared by a simple free-radical grafting polymerization of CMC and AM monomers in the O/W Pickering-MIPEs composed of non-toxic and low-cost vegetable oil as the dispersed phase and natural Pal as the stabilizer. The emulsions prepared under the optimum conditions (Pal content, 9–14%; T-20 content, 3%; AM dosage, 20 mmol) can act as efficient template to prepare a superporous polymer monolith presenting interconnected network. The introduction of small amount of T-20 surfactant contributes to improve the stability of the Pickering-MIPEs, which facilitate to form closed-cell porosity or highly interconnected small pores. The porous monoliths show high adsorption capacities of 1,585 and 1,625 mg/g for MV and MB, respectively, and fast adsorption rate. The porous monoliths can be regenerated by a simple desorption process. After five adsorption-desorption cycles, the regenerated monoliths still have the adsorption capacities of 1,108 and 1,257 mg/g for MV and MB, respectively. In a word, the eco-friendly Pickering-MIPEs composed of edible oil and natural Pal provide a new approach to fabricate high-efficient and recyclable porous monolith adsorbents for the decontamination of high-concentration dye wastewater.

## Author contributions

FW and YZ contribute to the experiment process and data analysis; wrote the paper and drawn all the figures. WW contributes to the design of experiment, data analysis, and revision of the paper. LZ and TL contribute to experiment process, structure characterization, and data analysis. AW contributes to the experiment design, data analysis, and revision of the paper.

### Conflict of interest statement

The authors declare that the research was conducted in the absence of any commercial or financial relationships that could be construed as a potential conflict of interest.
