# Supplementary material for: Fabrication of CMC-g-PAM Superporous Polymer Monoliths via Eco-Friendly Pickering-MIPEs for Superior Adsorption of Methyl Violet and Methylene Blue
Source: Front Chem. 2017 Jun 8;5:33. doi: 10.3389/fchem.2017.00033 (PMC5462918; doi:10.3389/fchem.2017.00033)
Supplement: Supplementary file 1 [file Table1.PDF]

Supplementary Material

Fabrication of CMC-*g*-PAM superporous polymer monoliths via eco-friendly Pickering-MIPEs for superior adsorption of methyl violet and methylene blue

Feng Wang[1,2], Yongfeng Zhu[1], Wenbo Wang[1,*], Li Zong[1], Taotao Lu[1,2], Aiqin Wang[1,*]

[1]Key Laboratory of Clay Mineral Applied Research of Gansu Province, Center of Eco-material and Green Chemistry, Lanzhou Institute of Chemical Physics, Chinese Academy of Sciences, Lanzhou 730000, P.R. China

[2]Graduate University of the Chinese Academy of Sciences, Beijing 100049, P.R. China

*** Correspondence:** aqwang@licp.cas.cn (A.Q. Wang); wbwang@licp.cas.cn (W.B. Wang)

Table S1. Adsorption isotherm constants for the adsorption of MV and MB onto porous PM monoliths.

Langmuir model								
Dyes	**MV**				**MB**			
Samples	$q_{e,\,exp}$ (mg/g)	q_m (mg/g)	b (L/mg)	R^2	$q_{e,\,exp}$ (mg/g)	q_m (mg/g)	b (L/mg)	R^2
PM-10	1031	996	0.0451	0.9989	1219	1153	0.0415	0.9958
PM-20	1219	1227	0.3484	0.9998	1327	1333	0.6158	0.9998
PM-30	1339	1350	0.3378	0.9997	1498	1505	0.4864	0.9999
PM-40	1479	1496	0.2826	0.9993	1562	1568	0.6104	0.9998
PM-50	1588	1602	0.3275	0.9995	1628	1636	0.4846	0.9997

Table S2 Adsorption isotherm constants for the adsorption of MV and MB onto porous PM monolith.

Freundlich model						
	MV			MB		
Samples	K	*n*	R^2	K	*n*	R^2
PM-10	170.08	3.23	0.8753	156.73	2.74	0.7399
PM-20	443.69	5.14	0.5607	561.70	5.88	0.4526
PM-30	483.16	4.96	0.5053	535.93	4.59	0.6320
PM-40	528.16	4.75	0.3911	612.75	4.94	0.4701
PM-50	530.62	4.22	0.4645	588.43	4.43	0.5177

Table S3 Adsorption kinetic parameters for adsorption of dyes onto the porous monoliths.

MV							
	Pseudo-first-order equation			Pseudo-second-order equation			
Samples	$q_{e,cal}$ (mg/g)	$K_1\times10^{-2}$ (min^{-1})	R^2	$q_{e,cal}$ (mg/g)	$K_2\times10^{-3}$ (g/mg min)	R^2	$q_{e,exp}$ (mg/g)
PM-10	144.0	2.81	0.2314	249.4	2.04	0.9981	245.8
PM-20	22.6	3.07	0.1607	248.9	4.71	0.9996	247.6
PM-30	9.0	3.66	0.2455	249.4	8.72	0.9999	247.1
PM-40	11.7	3.75	0.5767	249.7	5.01	0.9998	247.6
PM-50	22.7	5.52	0.4416	249.4	8.72	0.9999	249.1

Table S4 Adsorption kinetic parameters for adsorption of MB onto the porous monoliths.

MB							
	**Preudo-first-order equation**			**Preudo-second-order equation**			
Samples	$q_{e,cal}$ (mg/g)	$K_1 \times 10^{-2}$ (min^{-1})	R^2	$q_{e,cal}$ (mg/g)	$K_2 \times 10^{-3}$ (g/mg min)	R^2	$q_{e,exp}$ (mg/g)
PM-10	16.4	2.62	0.2940	250.15	2.32	0.9989	245.8
PM-20	6.9	2.0	0.2620	249.08	8.70	0.9998	248.1
PM-30	7.0	1.73	0.1508	247.74	7.85	0.9998	247.1
PM-40	4.7	1.16	0.1286	247.47	28.6	0.9999	247.9
PM-50	5.8	1.32	0.2129	248.38	20.9	0.9999	249.1
